# Changes in Quality and Safety Indexes During Rice Harvest and Discussion on Drying Technology

**DOI:** 10.3390/foods14071225

**Published:** 2025-03-31

**Authors:** Yujia Wang, Wenfu Wu, Jie Xu, Ming Gao, Zidan Wu, Rui Wang, Houqing Liu

**Affiliations:** 1 School of Biological and Agricultural Engineering, Jilin University, Changchun 130022, China; 2 Wilmar (Shanghai) Biotechnology Research & Development Center Co., Ltd., Shanghai 200137, China

**Keywords:** natural drying, mechanical drying, rice quality and safety indexes

## Abstract

This study investigated the effects of natural and mechanical drying on the quality and safety indices of newly harvested rice. The quality indices (moisture content, dry-basis 1000-grain weight, yellowing rate, gelatinization characteristics, and antioxidant enzyme activity) and safety indices (zearalenone, vomitoxin, and aflatoxin B1) were evaluated post-drying. The results demonstrated that natural drying significantly outperformed mechanical drying in terms of quality retention and antioxidant enzyme activity, particularly in preserving product integrity. In contrast, mechanical drying excelled in drying cost, speed, and process controllability. Using the Analytic Hierarchy Process (AHP), the quality and safety indices of rice dried by both methods were comprehensively assessed. The scoring results indicated that rice dried by natural methods had superior quality compared to that dried mechanically.

## 1. Introduction

Rice (*Oryza sativa* L.) is one of the most important food crops globally, particularly in Asia [1,2]. In 2024, China’s rice planting area reached 300 million hectares, with an output of 210 million tons, ranking first in both area and production worldwide [3]. Newly harvested rice typically has a high moisture content. If not dried promptly to below the safe moisture threshold, there may be adverse effects such as rice fever, germination, and mildew, resulting in significant losses [4,5,6].

Grain drying is a crucial step in post-harvest processing and equally important as increasing grain production [7,8]. Rice, being highly heat-sensitive, requires careful drying methods to prevent quality deterioration. Therefore, selecting appropriate drying methods is critical. Scientifically sound and efficient drying technologies can significantly reduce grain losses during storage and transportation, enhance both the quality and economic value of the grain, and optimize drying processes to ensure the quality and safety of the grain. This has become a key focus of current research [9,10].

Currently, the most common drying methods in China are natural and mechanical drying. Natural drying remains the primary method in some rural areas, while mechanical drying has become a key technology in modern rice processing. The existing literature primarily focuses on comparative analyses of rice drying time, energy consumption, and roughness rate across different drying methods. However, systematic research on rice quality—such as antioxidant enzyme activity, gelatinization characteristics, and toxin contamination—remains limited. Therefore, exploring the effects of natural and mechanical drying on the quality and safety indices of rice at harvest time holds significant theoretical and practical value.

In this experiment, two drying methods—natural drying and mechanical drying—were used to dry rice at harvest. The quality and safety indices of rice under both methods were tested, and the changes in water content, antioxidant enzyme activity, gelatinization characteristics, and toxin contamination were analyzed and compared. The objective was to assess the advantages and disadvantages of natural and mechanical drying technologies, providing insights for selecting effective and appropriate drying methods for post-harvest rice. Additionally, this study aims to offer new ideas and approaches for improving rice processing efficiency and reducing food safety risks.

## 2. Materials and Methods

### 2.1. General Situation of Experimental Site

The experiment was conducted at the Wuchang City experimental site (latitude 45.02°, longitude 127.37°), where the soil is classified as meadow black soil. The experimental field featured a deep soil layer, a convenient irrigation and drainage system, stable water and nutrient supply, and good permeability. During the growing season, crop management practices adhered to local agricultural standards, and there were no environmental stress factors.

### 2.2. Experimental Material

The experimental material used was Daohuaxiang 2, a japonica rice variety primarily cultivated in Wuchang City, Heilongjiang Province. The growth period of the tested material ranged from 138 to 140 days, classifying it as a late-maturing variety.

### 2.3. Experimental Method

#### 2.3.1. Drying Process

In this experiment, two drying methods—natural drying and mechanical drying—were employed.

##### Natural Drying

**Figure 1:**
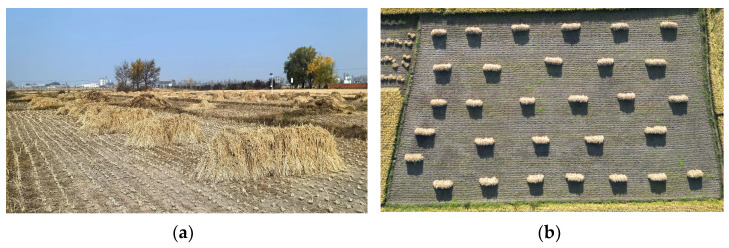
Natural drying experimental site: (**a**) front view; (**b**) top view.

The natural drying method involved bundling and stacking the rice, harvested either manually or mechanically, and drying it in the field. The water in the rice was evaporated due to wind, utilizing heat energy from sunlight and wind energy generated by natural climate conditions, thus reducing the moisture content in the rice, as shown in Figure 1. This method is simple and easy to operate; however, it requires a large airing area, has a long drying period, and is highly dependent on environmental factors. Additionally, it leads to increased impurity content, seed breakage, and uncontrollable moisture levels after drying that do not meet the requirements of modern industrial production [11].

##### Mechanical Drying

**Figure 2:**
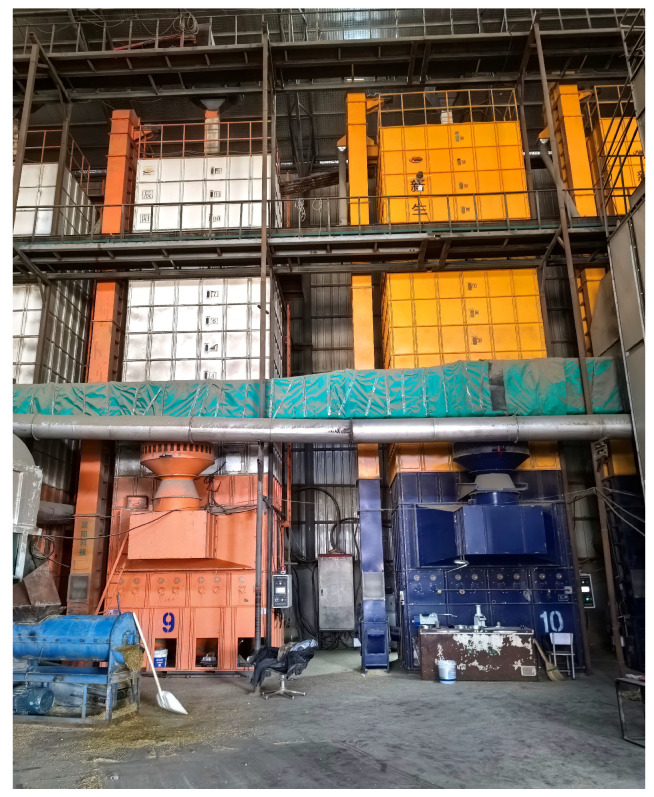
Mechanical drying experimental site.

The mechanical drying method utilized a batch-circulating grain dryer to dry rice, as shown in Figure 2. In this experiment, the hot air temperature was set to 60 °C, the variation range of the hot air velocity was set to 0.5 m/s~0.8 m/s, and the average precipitation rate was 0.35%/h, which finally reduced the moisture content of rice to 15%. A batch-circulating grain dryer is a type of equipment designed to reduce the moisture content of harvested grain, ensuring that it can be stored for extended periods without mildew or quality degradation. Its working principle is primarily based on the contact between hot air and grain, with excess moisture being removed through heating and ventilation. This method offers advantages such as short drying time, large capacity, and controllable drying temperature; however, it suffers from high energy consumption and uneven drying.

#### 2.3.2. Quality Index

##### Moisture Content

The moisture content (W_H_2_O_) of rice grains was determined by an accurate water detection method [12,13].

##### Dry-Basis 1000-Grain Weight

Approximately 500 rice grains were randomly selected using the quartering method. The complete grains were then separated and weighed (mₜ) and the number of complete grains (N) was recorded. The dry-basis 1000-grain weight (M) was subsequently calculated using formula (1), with the result accurate to 0.0001 g. All tests were repeated three times to obtain an average value. The formula was as follows:

(1) $$M=m_{t}\times 10\times \left(100-W_{H_{2}O}\right)÷N$$

##### Yellowing Rate

Representative samples were randomly selected from the field or stored rice, with each sample generally weighing no less than 500 g. The seeds were placed on a white background and observed visually under natural light or sufficient illumination. If the yellowing area of the grain exceeded half of the surface area and the entire grain appeared noticeably yellow, it was classified as yellowing. To minimize error, sampling and counting were repeated multiple times and the average value was used as the final result. The formula was as follows:

(2) $$YR\left(\%\right)=\frac{YN}{TN}\times 100$$

where YR is the yellowing rate, YN is the number of yellowed grains, and TN is the total number of grains.

##### RVA Characteristic Spectrum (Viscosity Characteristics of Rice)

First, the rapid viscosity analyzer was started and preheated for 30 min. Then, 15 g of rice was weighed, ground using a controllable tube grinder, and passed through a metal mesh screen with an aperture of 0.15 mm for further use. Next, 25 ± 0.1 mL of water was measured and transferred to a clean, dry sample tube (provided with the equipment). A 3 ± 0.01 g sample was weighed using a balance and transferred to the sample tube. It was stirred quickly with a stirrer to disperse the sample. If the sample remained suspended for more than 1 min, it was no longer used. Finally, it was kept at 50 °C for 1 min; then, the temperature was raised to 95 °C at a rate of 12 °C/min for 2.5 min and then lowered to 50 °C at the same rate for 2 min. The test process was automatically controlled by the instrument and the results were displayed on the screen. The experiment was repeated three times and the average of the results was recorded.

##### Antioxidant Enzyme Activity

In this experiment, peroxidase (POD), superoxide dismutase (SOD), and catalase (CAT) activity were measured using specific assay kits.

#### 2.3.3. Safety Index

The safety indices determined in this experiment included aflatoxin B1 (AFB1), vomitoxin (DON), and zearalenone (ZEN).

##### Determination of Aflatoxin B1

- Materials and Instruments

Reagents: Methanol and acetonitrile (chromatographic grade); other reagents (analytical grade); water (Grade I, as specified in GB/T 6682 [14]).

**Figure 3:**
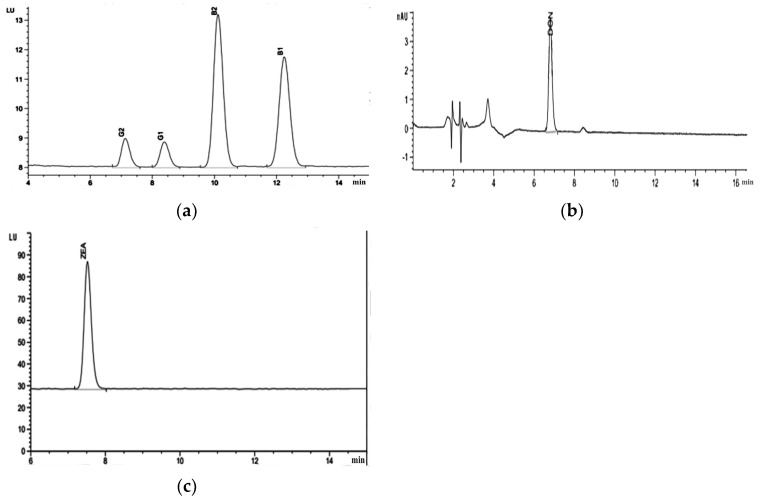
Standard chromatograms of mycotoxins: (**a**) represents the standard chromatogram of aflatoxins B1, B2, G1, and G2; (**b**) represents the standard spectrum of vomitoxin; (**c**) represents the standard chromatogram of zearalenone.

Standards: Aflatoxin standard solution (1.0 µg/mL, Romer). The standard spectrograms are shown in Figure 3a.

Columns: Immunoaffinity column for aflatoxin determination (Huaan Maike).

Equipment: High-performance liquid chromatograph equipped with a fluorescence detector and post-column derivatization system (Agilent 1260); solid-phase extraction device; centrifuge; analytical balance; vortex mixer.

- HPLC Reference Conditions

Column: Zorbax Eclipse XDB-C18 (4.6 × 150 mm, 5 µm).

Mobile Phase: Phase A: water; Phase B: methanol–acetonitrile (50:50, *v*/*v*).

Flow Rate: 1.0 mL/min.

Column Temperature: 40 °C.

Injection Volume: 50 µL.

Detection Wavelength: Excitation at 360 nm, emission at 440 nm.

- Sample Preparation

We weighed 5 g of sample (to 0.01 g) into a 50 mL centrifuge tube, added 20 mL of methanol–water (70:30, *v*/*v*), homogenized it for 3 min, and centrifuged it at 6000 rpm for 10 min. We then transferred 4 mL of the supernatant, added 23 mL of 1% Tween-20 PBS, and mixed it thoroughly.

- Purification

We equilibrated the immunoaffinity column to room temperature. We discarded the liquid, loaded the sample solution, and adjusted the flow rate to 1–3 mL/min. We washed the column twice with 10 mL water, dried it using a vacuum, and eluted it with 1 mL methanol. We collected eluates in a 10 mL tube, adjusted it to 3.0 mL with the mobile phase, vortexed it for 30 s, and filtered it through a 0.22 μm membrane for injection.

##### Determination of Vomitoxin

- Materials and Instruments

Reagents: Methanol, acetonitrile (chromatographic grade); other reagents (analytical grade); water (Grade I, GB/T 6682 [14]).

Standards: Vomiting toxin standard (100 µg/mL, Romer). The standard spectrum of deoxynivalenol (vomitoxin) is shown in Figure 3b.

Chemicals: Polyethylene glycol (MW: 8000).

Buffer: PBS buffer (8.0 g NaCl, 1.2 g Na_2_HPO_4_, 0.2 g KH_2_PO_4_, 0.2 g KCl in 990 mL water, pH 7.0 with HCl, diluted to 1 L).

Columns: Immunoaffinity column for emetic toxin (Huaan Maike).

Equipment: HPLC (UV detector); solid-phase extractor; centrifuge; analytical balance; vortex mixer.

- HPLC Reference Conditions

Column: Zorbax Eclipse XDB-C18 (150 mm × 4.6 mm, 5 µm).

Mobile Phase: Methanol–water (20:80, *v*/*v*).

Flow Rate: 0.8 mL/min.

Column Temperature: 35 °C.

Injection Volume: 50 µL.

Detection Wavelength: 218 nm.

- Sample Preparation

We weighed a 25 g sample (to 0.01 g) into a 250 mL flask, added 5 g polyethylene glycol and 100 mL water, mixed it thoroughly, and ultrasonicated/vortexed/shook it for 30 min. We then filtered it through quantitative and glass fiber filter paper until clear and centrifuged it at 10,000 rpm for 5 min.

- Purification

We mounted the column on the solid-phase extractor and attached a syringe. We discarded the liquid and then transferred 2 mL filtrate into the syringe. We adjusted the flow rate to 1–2 drops/s (d/s) until air passed through. We eluted it sequentially with 5 mL PBS buffer and 5 mL water at 1–2 d/s until air passed.

- Elution

We added 2.0 mL methanol for elution at a rate of 1 drop/s (d/s). We collected eluates in a centrifuge tube and dried them under nitrogen. We then reconstituted them with the mobile phase and analyzed them by HPLC-DAD.

##### Determination of Zearalenone

- Materials and Instruments

Reagents: Methanol, acetonitrile (chromatographic grade); other reagents (analytical grade); water (Grade I, GB/T 6682 [14]).

Standards: Zearalenone (C_18_H_22_O_5_) standard (100 µg/mL, Romer). The standard chromatogram of zearalenone is shown in Figure 3c.

Columns: Immunoaffinity column for zearalenone (Romer).

Equipment: HPLC (fluorescence detector); solid-phase extractor; centrifuge, analytical balance; vortex mixer.

- HPLC Reference Conditions

Column: Zorbax Eclipse XDB-C18 (4.6 × 150 mm, 4 µm).

Mobile Phase: Acetonitrile–water–methanol (46:46:8, *v*/*v*/*v*).

Flow Rate: 1.0 mL/min.

Column Temperature: Room temperature.

Injection Volume: 100 µL.

Detection Wavelength: Excitation at 274 nm, emission at 440 nm.

- Sample Preparation

We accurately weighed 40.0 g of sample (to 0.1 g) into a conical flask, added 4.0 g of sodium chloride and 100 mL of acetonitrile–water (90:10, *v*/*v*), and homogenized it at high speed for 1 min. We filtered the mixture through quantitative filter paper, transferred 10.0 mL of the filtrate, diluted it with 40.0 mL of water, and filtered it through a glass fiber filter until the filtrate was clear. The clear filtrate was reserved for subsequent use.

- Purification

We connected the immunoaffinity column to a 10 mL syringe, transferred 10.0 mL of sample extract, and passed it through at a flow rate of 1–2 drops per second (d/s) until air entered the column. We rinsed it sequentially with 10 mL of PBS/Tween-20 buffer and 10 mL of water at 1–2 d/s, discarding all effluent. We eluted it with 1.5 mL of methanol at approximately 1 d/s, collected the eluent in a clean test tube, and evaporated it to dryness under nitrogen below 55 °C. We dissolved the residue in 1.0 mL of the mobile phase for liquid chromatography analysis. We performed a blank test following the same procedure without the sample to confirm the absence of interfering substances.

#### 2.3.4. Comprehensive Scoring Method

The comprehensive evaluation values of rice seeds under different drying methods were obtained using the AHP analytic hierarchy process, with drying quality index, safety index, and drying cost as the weighted criteria based on their respective importance.

##### Establish a Hierarchical Model

**Figure 4:**
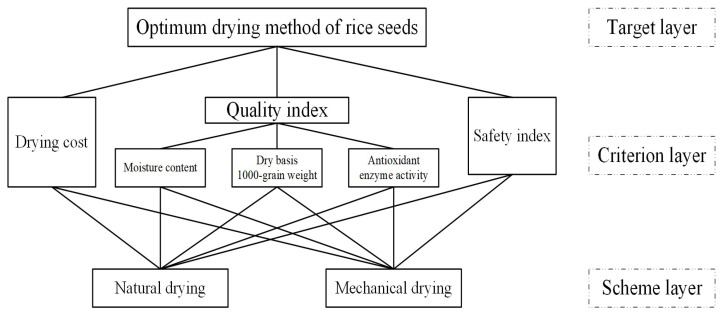
Hierarchical structure diagram.

In this study, the objective was to select the most suitable drying method for rice seeds, which was set as the goal layer. The criterion layer reflected the factors influencing the selection, with drying quality index, safety index, and drying cost as the first-level evaluation indices and moisture content, dry-basis 1000-grain weight, and antioxidant enzyme activity as the second-level evaluation indices. The scheme layer included the two drying methods as alternatives. Through these steps, the hierarchical structure of the evaluation scheme was constructed, as shown in Figure 4.

##### Construction of Judgment Matrix

After the hierarchical model was established, the 1–9 scale method and its reciprocal, as shown in Table 1, were used to assess the importance of each index in the criterion layer through pairwise comparison, thereby constructing a comparative judgment matrix [15].

In this study, the judgment matrix was constructed using the expert scoring method, where experts assess the relative importance of each factor based on relevant experience and data. The first-order judgment matrix (Table 2a) and the second-order judgment matrix (Table 2b) were derived using the average method.

##### Matrix Consistency Test

A matrix consistency test was performed (with CR < 0.1, indicating that the judgment matrix passed the consistency test) and the weights of each index were obtained (Table 3).

##### Data Standardization and Comprehensive Score Calculation

To standardize the data and ensure uniformity, the evaluation index data with different units were converted into a 0–1 scale. Among these, dry-basis 1000-grain weight and antioxidant enzyme activity were positive indicators and their standardization was calculated using Formula (3). Moisture content, safety index, and drying cost were negative indicators and their standardization was calculated using Formula (4).

(3) $$\begin{array}{c} S_{+}=\frac{C_{max}-C_{i}}{C_{max}-C_{min}} \end{array}$$

(4) $$S_{-}=\frac{C_{i}-C_{min}}{C_{max}-C_{min}}$$

where $C_{i}$, $C_{max}$, and $C_{min}$ are the measured values, maximum values, and minimum values of each index, respectively.

The calculation formula of the comprehensive score was as follows:

(5) $$\begin{array}{c} T=63.335\%\times \left(16.342\%\times S_{MC}+53.961\%\times S_{DW}+29.696\%\times S_{AE}\right)+26.050\%\times S_{SI}+10.616\%\times S_{DC} \end{array}$$

where S is the standardized data of the measured values of each index.

## 3. Results

### 3.1. Changes in Rice Grain Quality and Safety Indicators Under Natural Drying Conditions

#### 3.1.1. Changes in Moisture Content, Dry-Basis 1000-Grain Weight, and Yellowing Rate

**Figure 5:**
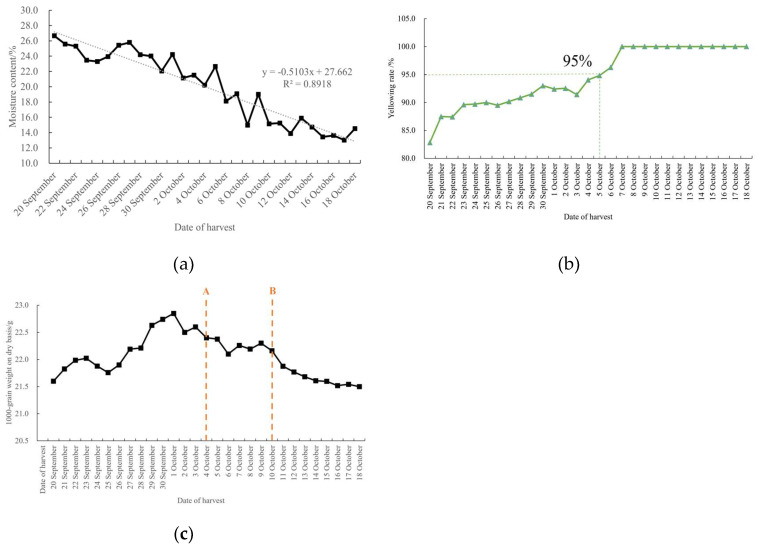
Variation curves of moisture content, dry-basis 1000-grain weight, and yellowing rate of rice under natural drying conditions: (**a**) variation curve of moisture content; (**b**) variation curve of yellowing rate; (**c**) variation curve of dry-basis 1000-grain weight.

Moisture content is a key factor influencing the physiological and biochemical processes of rice grains [16]. As shown in Figure 5a, the grain moisture content of Daohuaxiang 2 decreased gradually during different harvest periods. Frost occurred around October 4 and, after this time point, the rice yellowing rate reached the harvestable standard, as depicted in Figure 5b. The moisture content of the rice grains decreased significantly, ultimately reaching a safe moisture level.

As shown in Figure 5c, with the progression of the harvest period, the dry matter accumulation in rice grains increased during the early stages. On October 4th, the first frost occurred, and the dry matter accumulation plateaued, no longer increasing, and even began to decline in subsequent days. This decline represented a latent loss of rice dry matter, which was calculated to be 4.51%.

#### 3.1.2. Variation of RVA Characteristic Spectrum (Viscosity Characteristics of Rice)

RVA characteristic values are primarily expressed by pasting temperature [17], peak viscosity, minimum viscosity, final viscosity, breakdown (maximum viscosity–hot paste viscosity), and set-back (cold paste viscosity–maximum viscosity) [18,19,20].

**Figure 6:**
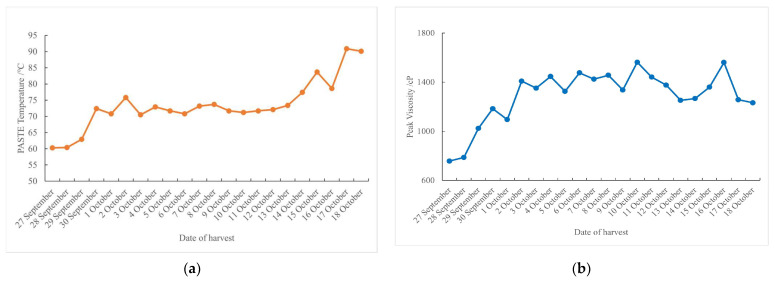

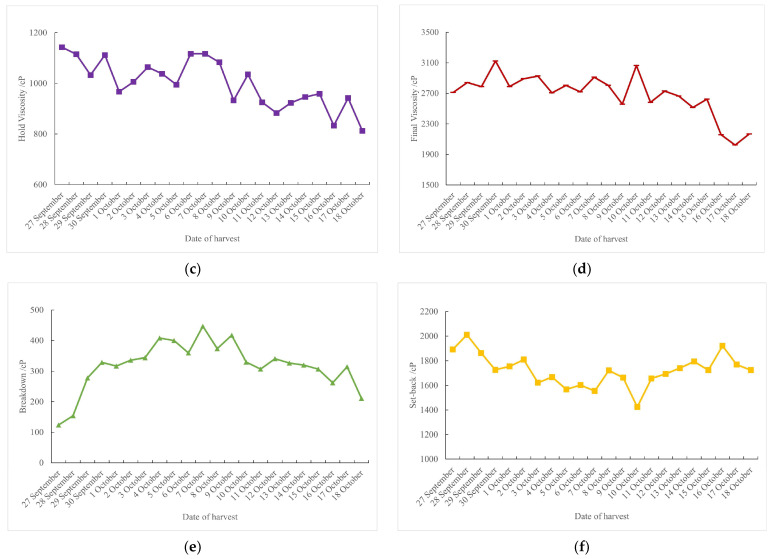
RVA characteristic spectrum (rice viscosity properties) curve under natural air-drying: (**a**) paste temperature; (**b**) peak viscosity; (**c**) minimum viscosity; (**d**) final viscosity; (**e**) collapse (the highest viscosity–hot paste viscosity); (**f**) setback (cold paste viscosity–maximum viscosity).

The pasting temperature refers to the lowest temperature at which starch begins to absorb water and swell to form a paste during heating. As shown in Figure 6a, the pasting temperature from October 3 to 13 was approximately 70 °C, remaining in a relatively stable range [21,22]—neither too high nor too low—and ensuring good cooking performance and edible quality.

Peak viscosity refers to the maximum viscosity reached by rice starch paste during the heating phase of rapid viscosity analysis (RVA). It is a key parameter for evaluating the cooking and eating quality of rice as it reflects the starch gelatinization behavior, which directly affects the texture and stickiness of cooked rice. During the harvesting period, the peak viscosity typically showed a trend of rising first and then falling. As shown in Figure 6b, the peak viscosity measured from October 2 to 12 was approximately 1400 cP, indicating a relatively stable period with minimal fluctuations in starch properties.

The lowest viscosity (also known as valley viscosity or retrogradation viscosity) refers to the minimum viscosity observed during the cooling of the rapid viscosity analysis (RVA) process, after the rice starch paste has reached peak viscosity. This value is influenced by starch retrogradation, which occurs as the temperature decreases. Rice harvested at the optimal time typically provides a moderate minimum viscosity, which is crucial for maintaining a balanced texture and taste after cooling. A moderate value indicates that the rice is neither too hard nor too soft, providing an appealing mouthfeel. As shown in Figure 6c, the minimum viscosity gradually decreases over time, likely due to starch retrogradation or changes in starch structure during cooling.

The final viscosity refers to the viscosity of rice starch paste measured at the end of the rapid viscosity analysis (RVA) process. In this study, the RVA profile was constructed by heating the sample from 50 °C to 95 °C at a rate of 12 °C/min, holding at 95 °C for 2.5 min, and then cooling back to 50 °C at the same rate. This process simulated the cooking and cooling of rice, with the final viscosity measured at 50 °C. A moderate final viscosity is crucial for maintaining the texture of rice during long-term storage as it reflects the stability of starch retrogradation. However, it should be noted that the final viscosity does not directly reflect rice’s ability to retain moisture, which is more closely associated with the peak viscosity observed during the heating phase. It ensures that cooked rice is neither too sticky, which may result in a clumpy texture, nor too dry and hard, which may lead to a poor texture. This balance contributes to a better taste and overall eating experience as it provides an appealing texture and consistency that is preferred by consumers. As shown in Figure 6d, the final viscosity gradually decreased during the harvesting period. This decline is likely attributable to changes in the starch structure, which may have affected the starch retrogradation process and, consequently, altered the final viscosity.

The disintegration value (also referred to as the disintegration temperature or disintegration characteristics) refers to the temperature range over which rice starch paste begins to absorb water, swell, and form a paste during the rapid viscosity analysis (RVA) process. It is a critical parameter for understanding the gelatinization behavior of starch. Specifically, it reflects the temperature range during which starch particles undergo swelling and paste formation. As shown in Figure 6e, during the harvesting period, the disintegration value of rice first increased and then decreased, reaching a maximum value of 447 cP on October 7.

Subtraction value (also referred to as the breakdown viscosity or retrogradation viscosity difference) refers to the difference between the peak viscosity and the lowest viscosity of rice starch paste during the rapid viscosity analysis (RVA). It reflects the stability of starch paste during heating and cooling and is an important indicator of starch retrogradation behavior. As shown in Figure 6f, during the harvesting period, the subtraction value of the rice first decreased and then increased, reaching a minimum value of 1425 cP on October 10.

#### 3.1.3. Changes in Antioxidant Enzyme Activity

Antioxidant enzyme activity is a crucial indicator of the vigor and stress resistance of rice seeds, reflecting the oxidative stress response and storage stability of rice during drying [23,24,25]. In this study, the activities of antioxidant enzymes in rice grains at various harvest stages were measured and analyzed, focusing primarily on peroxidase (POD), catalase (CAT), and superoxide dismutase (SOD) activity.

**Figure 7:**
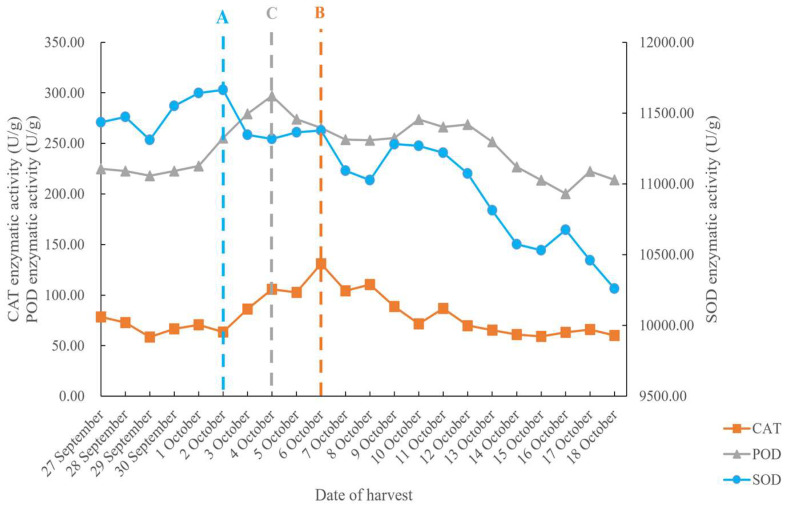
Activity curve of antioxidant enzymes under natural drying conditions: A is the highest SOD value; B is the highest CAT value; C is the highest POD value.

As shown in Figure 7, with the progression of the harvest period, the antioxidant enzyme activities in rice initially increased and then decreased. The activity of catalase (CAT) gradually rose to 130.89 U/g on October 6 and subsequently began to decline. Peroxidase (POD) activity increased to 296.99 U/g on October 4, then decreased, slightly increased again, and then continued to decline. Superoxide dismutase (SOD) activity exhibited significant fluctuations throughout the harvest period, with the highest value reaching 11,664.96 U/g on October 2. SOD, POD, and CAT, as key antioxidant enzymes in plants, play a critical role in scavenging excess free radicals, thereby enhancing plant stress resistance. The increase in enzyme activity suggests an enhancement in the antioxidant capacity and cellular activity, while the subsequent decrease in enzyme activity with prolonged harvest time indicates a decline in the plant’s resistance mechanism once the grain reaches a certain age, surpassing its regulation capacity [26,27,28].

#### 3.1.4. Changes in Mycotoxin Content

Mycotoxin contamination is a significant factor that threatens the safety of rice, including ZEN, DON, and AFB1. According to a survey conducted by the State Grain Bureau, the average loss rate of farmers’ stored grain in China is approximately 8%, resulting in an annual loss of about 40 billion kg [29]. The primary cause of this loss is mycotoxin contamination, accounting for about 30%. The highest detection rates are observed for ZEN [30], DON [31], and AFB1 [32]. The maximum allowable limits for these toxins in food raw materials and/or edible parts of food products are 60 μg/kg, 1 mg/kg, and 10 μg/kg, respectively [33].

In this experiment, three mycotoxins (ZEN, DON, and AFB1) were detected to assess contamination levels and trends in rice during drying. The results indicated that among the 22 samples collected under natural air-drying, the ZEN content in a sample from September 30th was 61 μg/kg, exceeding the maximum allowable limit for edible parts. No toxins were detected in the other samples, or their concentrations did not exceed the maximum allowable limit.

### 3.2. Changes in Rice Grain Quality and Safety Index Under Mechanical Drying Conditions

- The moisture content decreased from 28.25% (w.b.) to 15.61% (w.b.) during mechanical drying. The initial weight entering the tower was 15 tons and the final weight leaving the tower was 12 tons.
- During mechanical drying, the activity of antioxidant enzymes exhibited an overall downward trend, as shown in Table 4.
- No toxin contamination was detected in the samples collected during mechanical drying.
- The changes in the RVA characteristic spectrum (viscosity characteristics of rice) during mechanical drying are shown in Table 5.

### 3.3. Cost Calculation

Based on the actual investigation conducted in this experiment, the drying costs under both modes were calculated. The machinery and equipment costs, as well as the operating costs under the mechanical drying mode, were not considered for the time being, as shown in Table 6.

### 3.4. Comprehensive Score of Different Drying Methods of Rice Grains

The drying cost, safety index, and quality indices (moisture content, 1000-grain weight on a dry basis, antioxidant enzyme activity), which are critical factors in the drying process, were selected to comprehensively evaluate different drying methods for rice seeds. The score for natural drying was significantly higher than that for mechanical drying, as shown in Table 7.

## 4. Discussion

### 4.1. Comprehensive Comparison of Rice Grain Quality and Safety Index Under Different Drying Methods

In this study, natural drying and mechanical drying were systematically compared, considering drying time, quality indices, safety index, and drying cost. To present the comparison results more clearly, Table 8 summarizes the key indicators of both drying methods.

- **Drying time and energy consumption**: As shown in Table 8, the mechanical drying process took approximately 36 h, during which, the water content decreased from 28.25% to 15.61%. In contrast, natural drying took about 20 days, which was significantly longer than mechanical drying. This disparity emphasizes the time difference between the two methods. Although natural drying does not require additional energy consumption, its prolonged drying period may negatively impact rice quality, particularly in high-humidity weather.
- **Antioxidant enzyme activity:** During the natural air-drying process, antioxidant enzyme activity initially increased and then decreased. In contrast, antioxidant enzyme activity during mechanical drying steadily decreased, suggesting that a reduction in enzyme activity in rice seeds indicates the onset of seed aging. As a result, the cells’ ability to remove peroxides diminished, ultimately leading to a decline in the activity of rice seeds.
- **Toxin pollution:** No toxins were detected in the samples collected during natural air-drying and mechanical drying or their content did not exceed the maximum limit.
- **RVA characteristic spectrum:** Table 8 also illustrates the differences in the gelatinization characteristics between the two drying methods. The pasting temperature and peak viscosity of the samples treated by natural drying were higher than those treated by mechanical drying, suggesting that natural drying may better preserve the native structure of rice starch, leading to improved gelatinization properties. As a result, natural drying may have more advantages in retaining the nutritional components and sensory characteristics of rice, which contributes to a softer and stickier texture in cooked rice. In contrast, mechanical drying may cause partial damage to the starch structure, resulting in lower pasting temperature and peak viscosity as well as potential losses in nutritional quality and sensory characteristics.
- **Cost-effectiveness:** Under mechanical drying, the cost per kilogram of rice was approximately CNY 0.4, while, under natural drying, it was about CNY 0.7. Compared to mechanical drying, natural drying is more costly.

### 4.2. Comprehensive Score Analysis of Rice Grains Under Different Drying Methods

In this study, drying cost, safety index, and quality indices (moisture content, 1000-grain weight on a dry basis, antioxidant enzyme activity) were selected as key factors in the drying process, and the different drying methods for rice seeds were comprehensively evaluated. The ranking of the comprehensive scores was as follows: natural drying > mechanical drying. Natural drying has clear advantages in drying quality and antioxidant enzyme activity, particularly in maintaining product quality. However, the low cost, rapid drying ability, and high controllability of mechanical drying make it more competitive for large-scale production.

Therefore, the choice of drying method should be based on the specific production needs and objectives. If the focus is on preserving the quality of rice during production, natural drying is undoubtedly the better option. However, if cost control, drying efficiency, and production speed are prioritized, mechanical drying is the more suitable choice. Overall, natural drying is ideal for small-scale, quality-focused production, while mechanical drying is better suited for large-scale, cost-effective production.

## 5. Conclusions

In this study, drying time, quality indices, safety indices, and cost were comprehensively analyzed through a systematic comparison of natural drying and mechanical drying methods. The results revealed that the quality and safety of rice seeds were significantly influenced by the drying method used.

Although natural drying excels at preserving rice quality, antioxidant enzyme activity, and nutritional components, its extended drying time and high labor input result in higher costs, which can lead to higher prices and reduce consumer demand in the early stages. In contrast, while mechanical drying offers a shorter drying time and lower costs, enhancing production efficiency and providing clear advantages in the cost control and production cycle, it is slightly less effective than natural drying in maintaining antioxidant enzyme activity and some quality indices.

In conclusion, rice drying is a complex process influenced by various factors. Choosing an appropriate drying method not only impacts rice quality and safety but also affects the economic interests of farmers. Therefore, the selection of rice drying technology should be tailored to specific production needs and objectives. For production systems focused on preserving rice quality and minimizing nutrient loss, natural drying is recommended. For large-scale enterprises that prioritize cost-effectiveness and production efficiency, mechanical drying is a more suitable choice.

## Figures and Tables

**Table 1:** Scale meanin of AHP from 1 to 9.

Relative Importance	Scale Value
Extremely important	9
Very important	7
Obviously important	5
Slightly important	3
Equal importance	1
Mean value	2,4,6,8
Exact inverse value	Reciprocal

**Table 2:** First-level judgment matrix (**a**) and second-level judgment matrix (**b**).

**(a)**			
**Item**	**Quality Index**	**Safety Index**	**Drying Cost**
Quality index	1	3	5
Safety index	1/3	1	3
Drying cost	1/5	1/3	1
**(b)**			
**Item**	**Moisture** **Content**	**Dry-basis** **1000-Grain Weight**	**Antioxidant** **Enzyme Activity**
Moisture content	1	1/3	1/2
Dry-basis 1000-grain weight	3	1	2
Antioxidant enzyme activity	2	1/2	1

**Table 3:** Matrix consistency test and weights of indicators.

Item	Proper Vector	Weight Value	Maximum Eigenvalue	CI Value
Quality index	1.900	63.335%	3.039	0.019
Safety index	0.781	26.050%		
Drying cost	0.318	10.616%		
Consistency check	CR = 0.04 and the consistency test passed.			
Moisture content	0.550	16.342%	3.009	0.005
Dry-basis 1000-grain weight	1.817	53.961%		
Antioxidant enzyme activity	1.000	29.696%		
Consistency check	CR = 0.06 and the consistency test passed.			

**Table 4:** Changes in antioxidant enzyme activity during mechanical drying.

	SOD	CAT	POD
Before (U/g)	10902.16	79.12	390.37
After (U/g)	10714.62	48.53	335.11

**Table 5:** Changes in the RVA characteristic spectrum during mechanical drying.

	Paste Temperature/°C	Peak Viscosity/cP	Minimum Viscosity/cP	Final Viscosity/cP	Collapse Value/cP	Subtraction Value/cP
Before	75.3	1238	943	2662	311	1719
After	90	1174	863	2523	295	1660

**Table 6:** Costs for natural drying and mechanical drying mode.

	Natural Drying		Mechanical Drying
Harvesting	Manually harvest CNY 360 per mu.	Harvester bales CNY 200 per mu.	Combine harvester CNY 150 per mu. (including harvesting, threshing, etc.)
	Stacking CNY 40 per mu.		
	Threshing CNY 240 per mu.		
Drying	No fees		Drying per ton CNY 160 (calculated by wet grain)
Total	CNY 0.9/kg	0.7 CNY/kg	0.4 CNY/kg

**Table 7:** Comprehensive scoring table for different drying methods of rice grains.

Drying Methods	Comprehensive Score
Natural drying	0.75
Mechanical drying	0.40

**Table 8:** Comparison of indexes between natural drying and mechanical drying.

Item		Natural Drying	Mechanical Drying
Moisture content		Drop from 26.7% to 16.5%	Drop from 28.25% to 15.61%
Drying time		About 20 days	About 36 h
Antioxidant enzyme activity		The overall trend is to rise first and then decline	The overall trend is downward
SOD enzyme activity (U/g)		11,435.35-11,664.96-10,260.22	10,902.16-10,714.62
POD enzyme activity (U/g)		224.94-296.99-214.04	390.37-335.11
CAT enzyme activity (U/g)		78.47-130.89-60.10	79.12-48.53
Toxin contamination situation		Except for the samples collected on September 30th, no toxins were detected	No toxins were detected
RVA characteristic profile	Paste temperature (°C)	60.3-70-90.1	75.3-90
	Peak viscosity (cP)	757-1400-1232	1238-1174
	Minimum viscosity (cP)	1143-812	943-863
	Final viscosity (cP)	3123-2023	2662-2523
	Breakdown value (cP)	124-447-211	311-295
	Setback value (cP)	2011-1425-1725	1719-1660
Cost (CNY/kg)		0.7-0.9	0.4

## Data Availability

The original contributions presented in this study are included in the article; further inquiries can be directed to the corresponding author.
